# Efficacy and safety of tranexamic acid in cervical spine surgery: a systematic review and meta-analysis

**DOI:** 10.3389/fneur.2024.1405773

**Published:** 2024-05-06

**Authors:** Hua Luo, Yu Yang, Zhitao Wang, Lingping Ma, Chengxin Xie

**Affiliations:** ^1^Department of Orthopedic, Taizhou Hospital of Zhejiang Province Affiliated to Wenzhou Medical University, Taizhou, Zhejiang, China; ^2^Department of Pharmacy, Taizhou Hospital of Zhejiang Province Affiliated to Wenzhou Medical University, Taizhou, Zhejiang, China; ^3^Department of Operation Room, Taizhou Hospital of Zhejiang Province Affiliated to Wenzhou Medical University, Taizhou, Zhejiang, China

**Keywords:** tranexamic acid, blood loss, cervical, complications, meta-analysis

## Abstract

**Background:**

Tranexamic acid (TXA) is an antifibrinolytic drug associated with reduced blood loss in a range of surgical specialties. This meta-analysis aimed to compare the efficacy and safety of TXA in cervical surgery, focusing on its effects on intraoperative blood loss and related outcomes.

**Methods:**

We searched the PubMed, EMBASE, Medline, and Cochrane Library databases to identify all literature related to TXA used in cervical spinal surgery. Intraoperative blood loss, postoperative drainage volume, total blood loss, postoperative hematological variables, and complications were analyzed.

**Results:**

Eight trials met the inclusion criteria. The pooled results showed that intraoperative blood loss, total blood loss, and postoperative drainage volume were significantly lower in the TXA group than in the control group. The hemoglobin and hematocrit on postoperative day 1 was significantly higher in the TXA group than in the control group. There was no significant difference in complications between the two groups.

**Conclusion:**

The available evidence indicates that TXA effectively reduces blood loss in cervical spinal surgery while maintaining a favorable safety profile, without increasing associated risks.

**Systematic review registration:**

https://www.crd.york.ac.uk/prospero/, identifier CRD42023459652.

## Introduction

Tranexamic acid (TXA) is a well-established antifibrinolytic agent that has been shown to reduce blood loss during joint replacement, cardiac surgery, and spine surgery (1–3). In spine surgery, perioperative bleeding can have serious consequences, including spinal cord injury and compression of the esophagus and trachea by hematoma (4). To reduce surgical blood loss and the associated morbidity and mortality, researchers have been exploring a variety of strategies, including administration of erythropoietin to promote preoperative hematocrit, intraoperative autologous transfusion, controlled hypotension, and isovolumetric hemodilution (5). However, these methods are not without risks and complications, and in some cases may not meet the requirements for cost-effectiveness. Therefore, there has been a focus on the potential of hemostatic agents, such as desmopressin, aprotinin, and others, to reduce blood loss and the need for transfusions (6). Unfortunately, desmopressin has not been shown to be effective, and aprotinin has not been cost-effective. Furthermore, the safety of antifibrinolytic agents remains uncertain, and previous studies have found that they are associated with an increased incidence of thromboembolic events (7). For these reasons, TXA is being studied as an alternative antifibrinolytic agent as one of the attempts to reduce blood loss during spinal surgery. In the normal fibrinolytic pathway, plasmin binds to fibrin through its lysine binding site and then undergoes fibrin degradation through its serine protease activity. TXA is a lysine analog that significantly reduces lysis of fibrin by competitively blocking lysine binding sites and inhibiting the activity of tissue plasminogen activator, plasminogen (a plasmin precursor), and plasmin (8). Therefore, TXA reduces degradation of platelets and promotes clot formation, thereby reducing the amount of blood lost during surgery. Previous systematic reviews and meta-analyses have shown that TXA can reduce blood loss in a variety of spine surgeries, including posterior lumbar interbody fusion, correction of spinal deformities in adults, and multilevel spine surgery (2, 9–13). However, to the best of our knowledge, there have been no meta-analyses of the efficacy of TXA in cervical surgery. In this meta-analysis and review, we present data from eight studies that investigated the safety and value of TXA in patients undergoing cervical surgery. The aim of this research was to further evaluate the effect of TXA on perioperative blood loss and complications, to update the medical evidence base, and to further clarify the specific role of TXA in cervical surgery so as to provide a reference for clinical work and accelerate the recovery of patients.

## Methods

According to the PRISMA (Preferred Reporting Items for Systematic Reviews and Meta-Analyses) statement, this meta-analysis was performed in agreement (14). The protocol for this meta-analysis was registered on PROSPERO (Registration No: CRD 42023459652).

### Inclusion criteria

Study type: randomized controlled trial (RCT), cohort study or case–control study. Study population: patients undergoing cervical surgery. Intervention and control: TXA used in the treatment group, no-TXA in the control group. Outcome index: intraoperative blood loss (IBL), postoperative drainage, total blood loss (TBL), postoperative hemoglobin (HB) and hematocrit (HCT) 1 day after surgery, and complications.

### Exclusion criteria

Letters, case reports, meeting, reviews, animal trials, or republished studies; TXA wasn’t used topically and intravenously in the treatment group; Studies lacking a control group; Patients with a past medical history of coagulopathy, bleeding disorders, seizures, blood clots.

### Search strategy

One of the authors performed the search in PubMed, EMBASE, Web of Science (Medline), and the Cochrane Central Register of Controlled Trials from the inception dates to 30 October 2023, using the keywords “(tranexamic acid or transexamic acid or TXA or Ttxa or t-amcha or amcha or cyklokapron or transamine) and (cervical or spinal or spine or vertebra or vertebrae) and (blood loss or complication or drainage or hemoglobin or HB or hematocrit or HCT).” No language restrictions were applied during the search.

### Study selection

Two researchers individually screened the retrieved literature strictly against inclusion and exclusion criteria. First, the documents that meet the inclusion criteria are read in full by reading the title and abstract, and the included papers are finally confirmed. If two researchers do not agree during the literature screening process, it will be left to the senior researcher.

### Data collection process

Data on relevant outcome measures that met the inclusion criteria were extracted from the literature, including author year, study design type, country, sample size, participants, TXA treatment, age, outcomes, etc.

### Assessment of risk of bias and quality of evidence

Two researchers independently assessed the quality of all included trials based on Cochrane risk-of-bias criteria (15). The Newcastle–Ottawa scale (NOS) was used to evaluate the literature quality of the retrospective studies (16).

### Data synthesis

The Meta-analysis was performed using Stata (version 17; StataCorp, 2021) software. The heterogeneity was assessed by using the Q test and I^2^ value calculation. The random effects model was used. The odds ratio (OR) and their associated 95% confidence interval (CI) were used to assess outcomes for dichotomous outcomes. Continuous outcomes were analyzed using mean, SD, and sample size to provide a mean difference (MD) between the TXA and control groups. A *p*-value less than 0.05 suggested that the difference was statistically significant.

### Sensitivity analyses

We performed a sensitivity analysis by excluding the largest trial, excluding trials with a high risk of bias.

## Results

**Figure 1:**
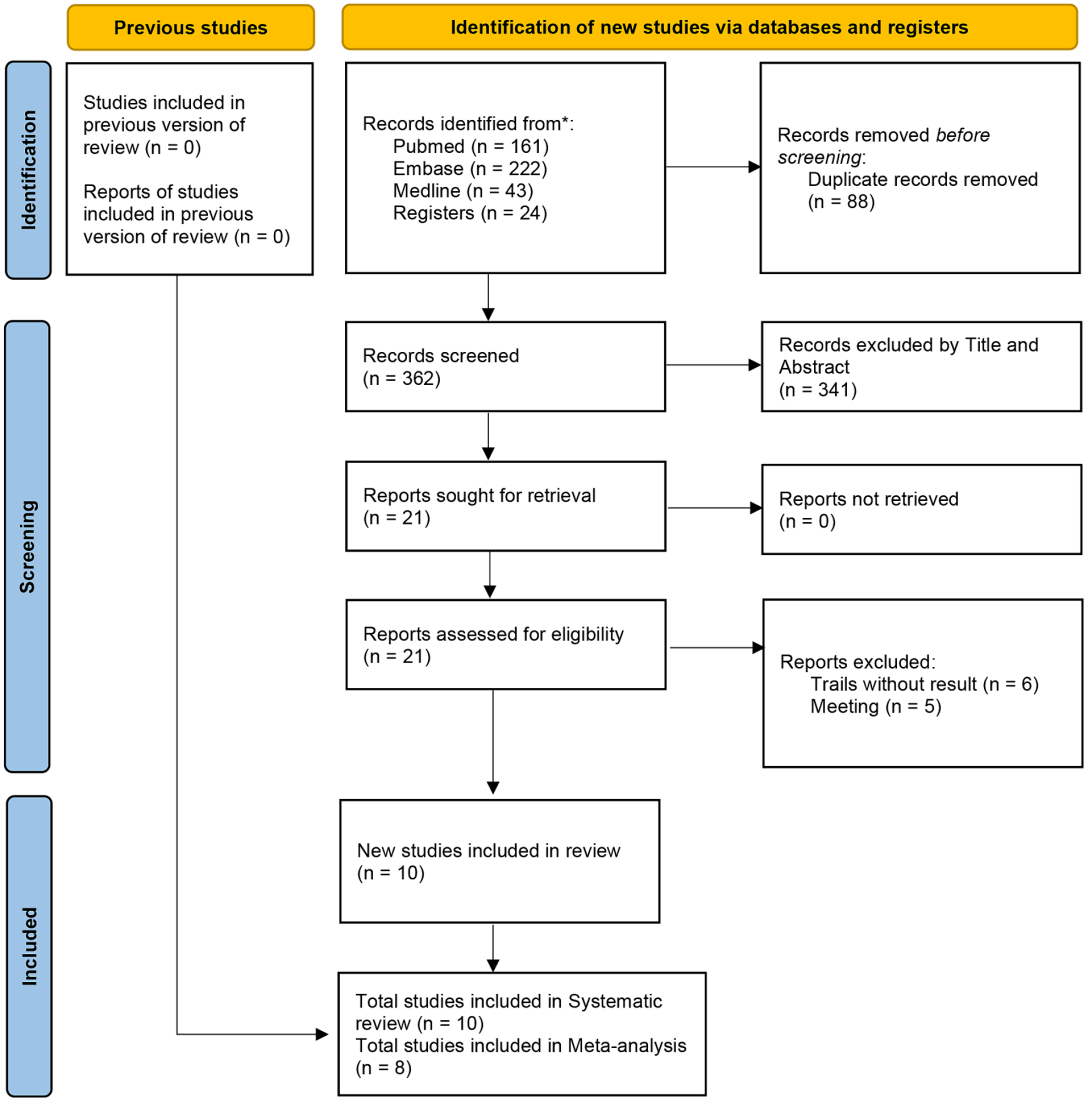
Flow diagram for search and selection of included studies.

The literature search yielded a total of 450 studies, 88 of which were duplicate publications and 341 were found to be irrelevant based on the titles and abstracts. After these articles were excluded, the full-text versions of 21 articles were read. Thirteen further articles were eliminated, including six trials that did not present results and five that were reports of meetings. Two trials included surgery of the spine, and it was not possible to extract data on the cervical spine, so we only included them in the review (17, 18). Finally, eight trials (two RCT and six retrospective studies including a total of 932 patients) (19–26) were included in the meta-analysis. The literature screening process is shown in Figure 1.

### Characteristics of included studies

The basic characteristics of the included studies are shown in Table 1. These studies were published between 2011 and 2023. Three studies originate from China, one from Hong Kong, two from the USA, one from Japan, and one from Iran. As illustrated in Table 2, Ma et al. (23) exhibited some bias overall due to unclear detection bias. Tsutsumimoto et al. (20) demonstrated high bias overall as a consequence of employing medical record numbers in the randomization process. All non-randomized studies received NOS of 8 points or higher, indicating high-quality research (see Table 3).

**Table 1:** Characteristics of included studies.

Study	Country	Participants	Treatment with TXA	Design	Age		Sex (F/M)		BMI		Outcomes	No. of subject	
					TXA	Control	TXA	Control	TXA	Control		TXA	Control
Chen, 2022	China	Degenerative cervical myelopathy	Gelfoam soaked with 1 g TXA	Retrospective cohort	64.4 ± 8.68	65.12 ± 8.72	11/33	9/36	22.14 ± 1.90	21.37 ± 2.27	IBL, volume and length of drainage, transfusions rate, length of hospital stay, hematological parameters, complications	44	45
			1 g TXA injected into the wound		63.8 ± 9.43		9/35		21.44 ± 2.18			44	
Ho, 2020	Hong Kong	Multilevel compressive cervical myelopathy	10 mg/kg iv TXA + maintenance 1 mg/kg/h iv TXA	Retrospective cohort	61 ± 9	63 ± 10	12/18	15/17	NA	NA	IBL, volume of drainage, hematological parameters, complications	30	32
Khadivi, 2023	Iran	Posterior cervical laminectomy and lateral mass screw fixation	3 g TXA irrigated	Retrospective cohort	51.1 ± 11.0	53.5 ± 13.7	23/25	18/22	28.3 ± 5.3	28.6 ± 5.7	IBL, volume of drainage, length of postoperative hospital, complication	48	40
Ma, 2022	China	Cervical spondylotic myelopathy	15 mg/kg iv TXA	RCT	62.76 ± 5.83	63.65 ± 7.45	19/36	18/37	25.39(22.58, 27.39)	26.21 (23.05, 28.08)	IBL, hematological parameters, volume and length of drainage, length of hospital stay, complication	55	55
			30 mg/kg iv TXA		62.34 ± 7.02		19/36		25.95(22.98, 27.85)			55	
Perez-Roman, 2019	USA	Cervical stenosis	10 mg/kg iv TXA + 1 mg/kg/h iv TXA	Retrospective cohort	60 ± 12	65 ± 12	7/12	13/7	NA	NA	TBL, IBL, complication	19	20
Steinle, 2023	USA	Anterior cervical discectomy and fusion	30 mg/kg iv TXA+ 3 mg/kg/h iv TXA	Retrospective cohort	54.25 ± 11.28	52.53 ± 11	52/44	89/101	30.25 ± 6.5	31.36 ± 7.63	IBL, volume and length of drainage, hematological parameters, transfusions rate, complications	96	190
Tsutsumimoto, 2011	Japan	Cervical multilevel compressive myelopathy	15 mg/kg iv TXA	RCT	68.0 ± 11.0	65.8 ± 11.8	4/16	5/15	23.7 ± 2.5	23.1 ± 2.4	IBL, TBL, volume of drainage, complication	20	20
Yu, 2017	China	Multilevel cervical spondylotic myelopathy	15 mg/kg iv TXA + 100 mg/h iv TXA	Retrospective cohort	64.4 ± 9.13	63.7 ± 8.85	11/62	9/37	21.42 ± 1.92	21.35 ± 2.3	IBL, TBL, hematological parameters, complication	73	46

RCT, randomized controlled trial; TXA tranexamic acid; IBL, intraoperative blood loss; TBL, total blood loss; NA, not applicable.

**Table 2:** Risk of bias assessment with the Cochrane assessment tool.

Author	Bias from randomization	Bias from allocation	Bias from performance	Bias from detection	Bias from attrition	Bias from reporting	Bias from other	Overall risk of bias
Ma, 2022	Low	Low	Low	Some	Low	Low	Low	Some
Tsutsumimoto, 2011	High	Some	Some	Some	Low	Low	Low	High

**Table 3:** New Castle–Ottawa scale ratings.

Study	Selection	Comparability	Exposure/outcome	Total score
Chen, 2022	****	**	***	9
Ho, 2020	****	**	***	9
Khadivi, 2023	****	**	***	9
Perez-Roman, 2019	****	**	***	9
Steinle, 2023	****	**	***	9
Yu, 2017	****	*	***	8

One * means score 1.

### Intraoperative blood loss

**Figure 2:**
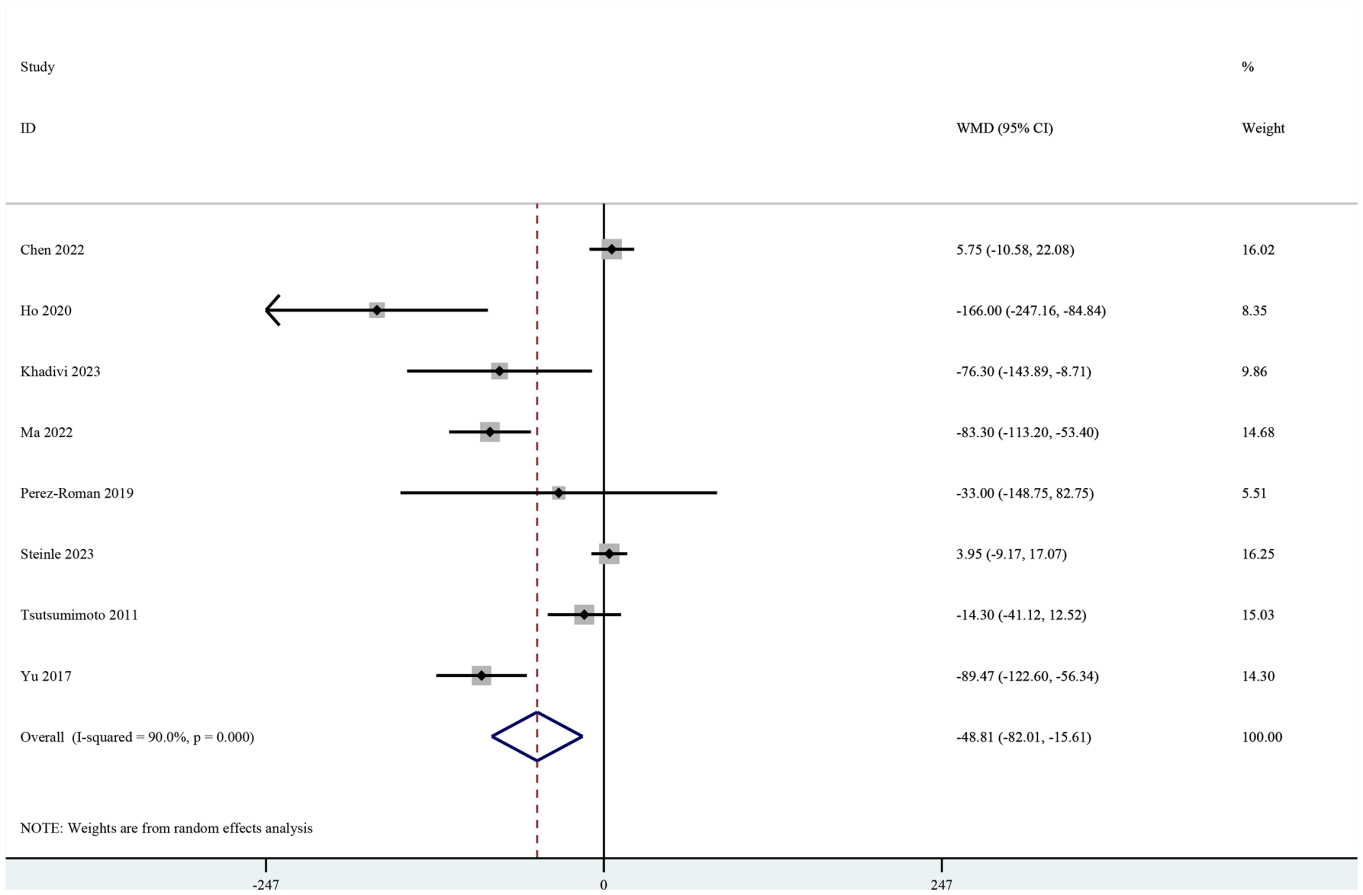
Forest plot of comparison: TXA vs. no-TXA; outcome: Intraoperative blood loss.

Eight studies included intraoperative blood loss as the primary outcome (19–26). The pooled results showed a significant reduction in intraoperative blood loss in the TXA group (MD-48.81, 95% CI-82.01, −15.61, I^2^ = 90.0%, *p* = 0.004; Figure 2).

### Total blood loss

**Figure 3:**
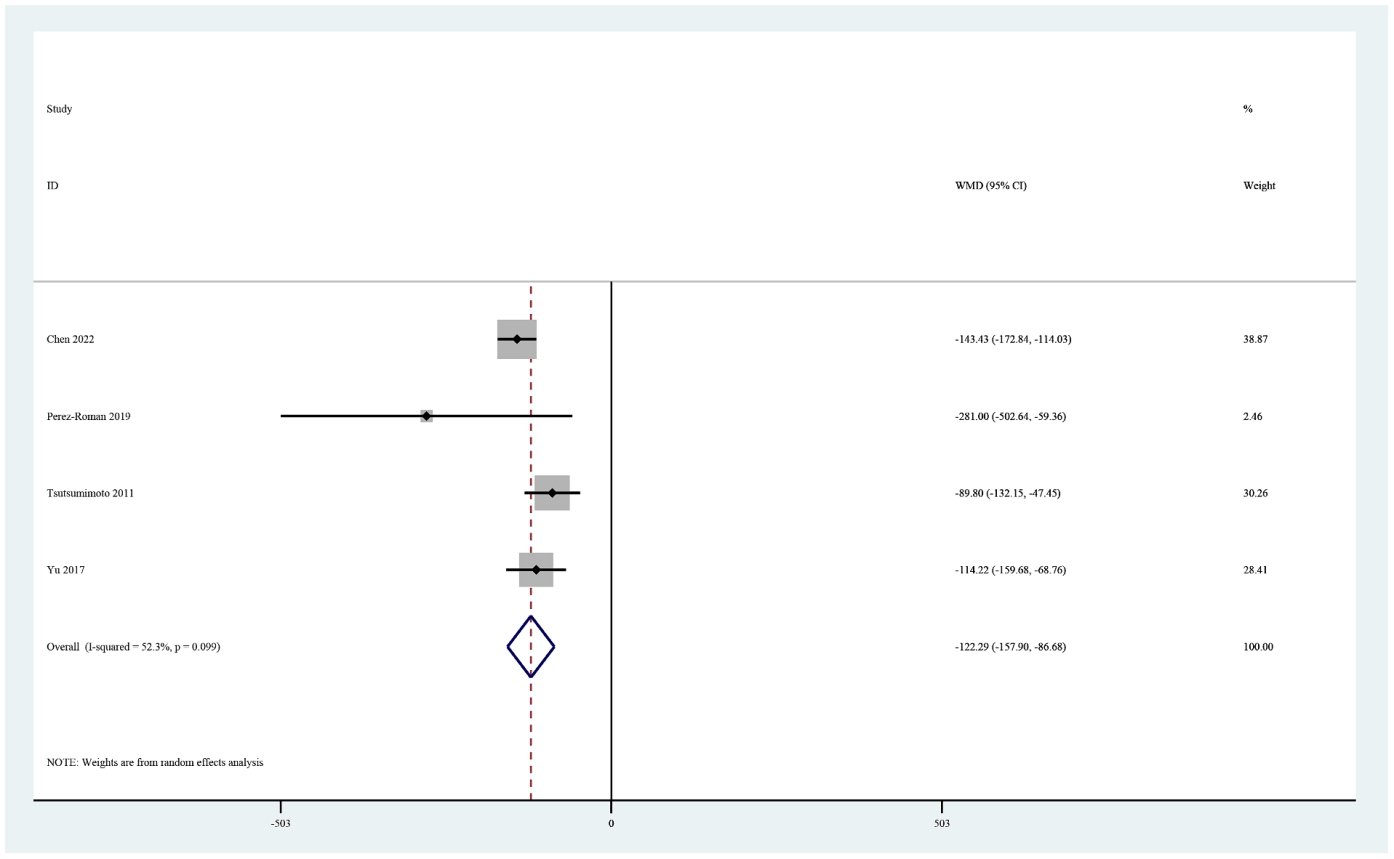
Forest plot of comparison: TXA vs. no-TXA; outcome: Total blood loss.

Four studies reported total blood loss (19, 20, 22, 26). In a random-effects model, the total amount of blood loss was significantly lower in the TXA group than in the control group (MD −122.29, 95% CI -157.90, −86.69, I^2^ = 52.3%, *p* < 0.0001; Figure 3).

### Postoperative drainage

**Figure 4:**
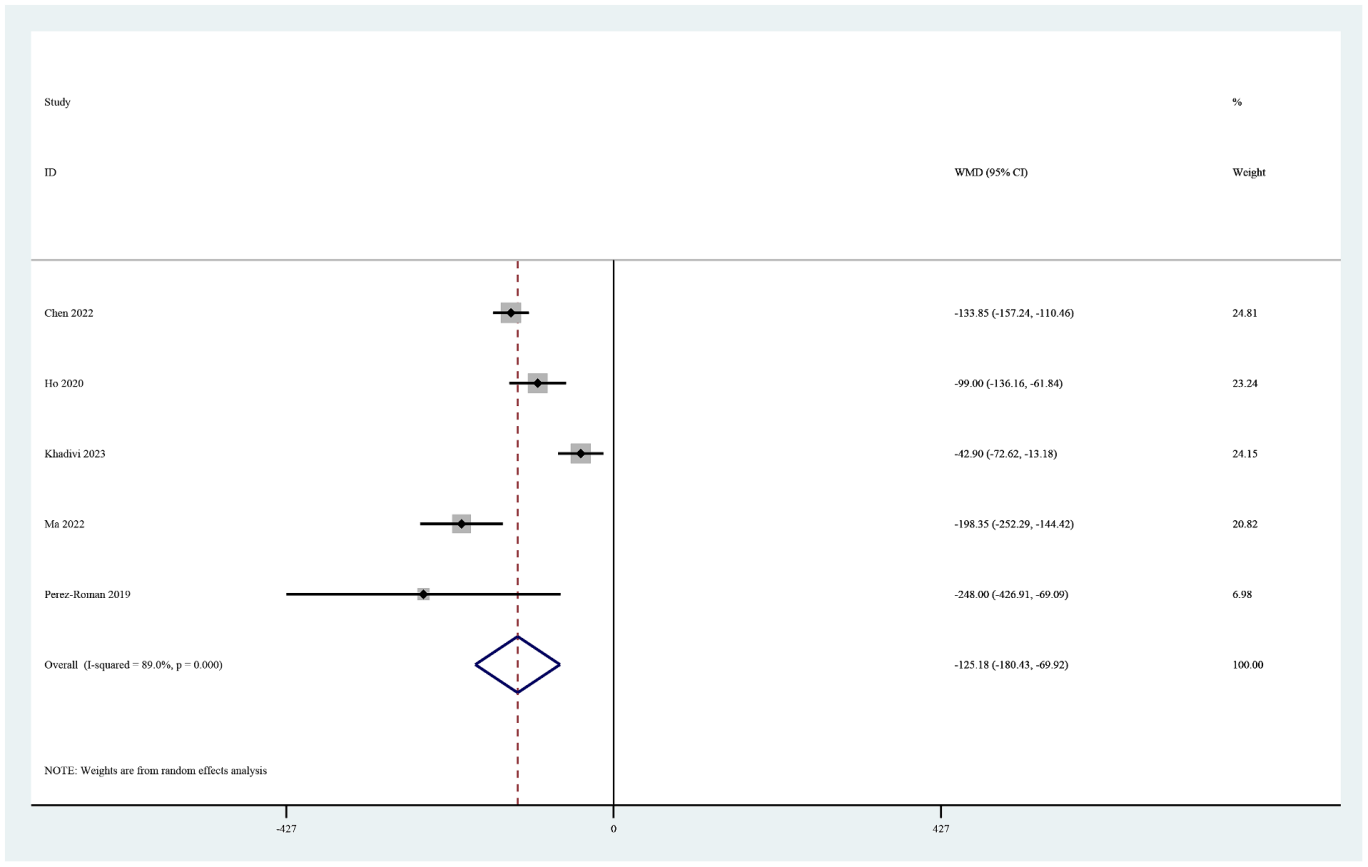
Forest plot of comparison: TXA vs. no-TXA; outcome: Postoperative drainage.

Five studies reported on postoperative drainage (22–26). Use of TXA in cervical spine surgery significantly reduced postoperative drainage (MD −125.18, 95% CI -180.44, −69.92, I^2^ = 89.0%, *p* < 0.0001; Figure 4).

### Postoperative hematological parameters on postoperative day 1

**Figure 5:**
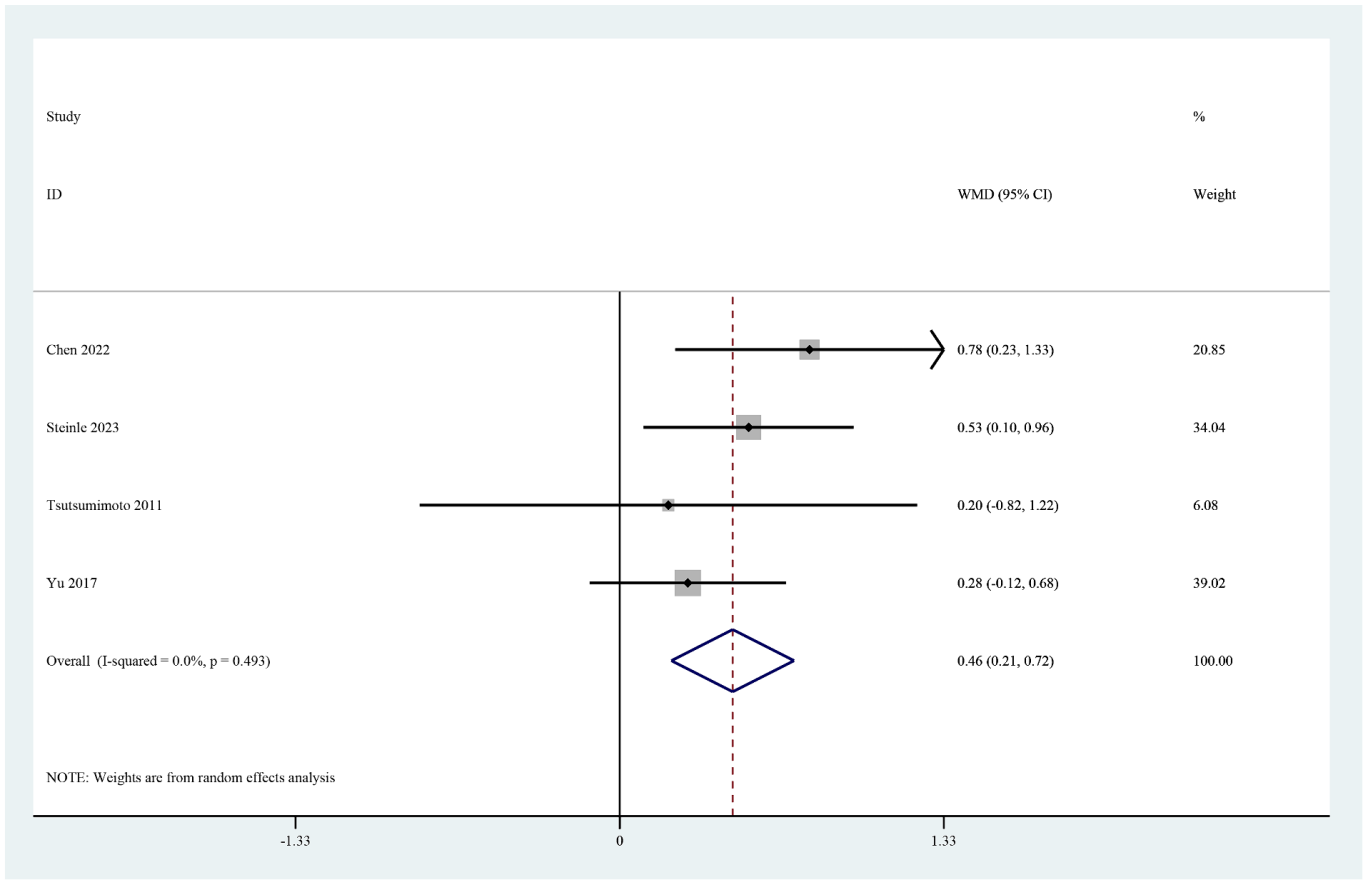
Forest plot of comparison: TXA vs. no-TXA; outcome: Hemoglobin levels on the first postoperative day.

**Figure 6:**
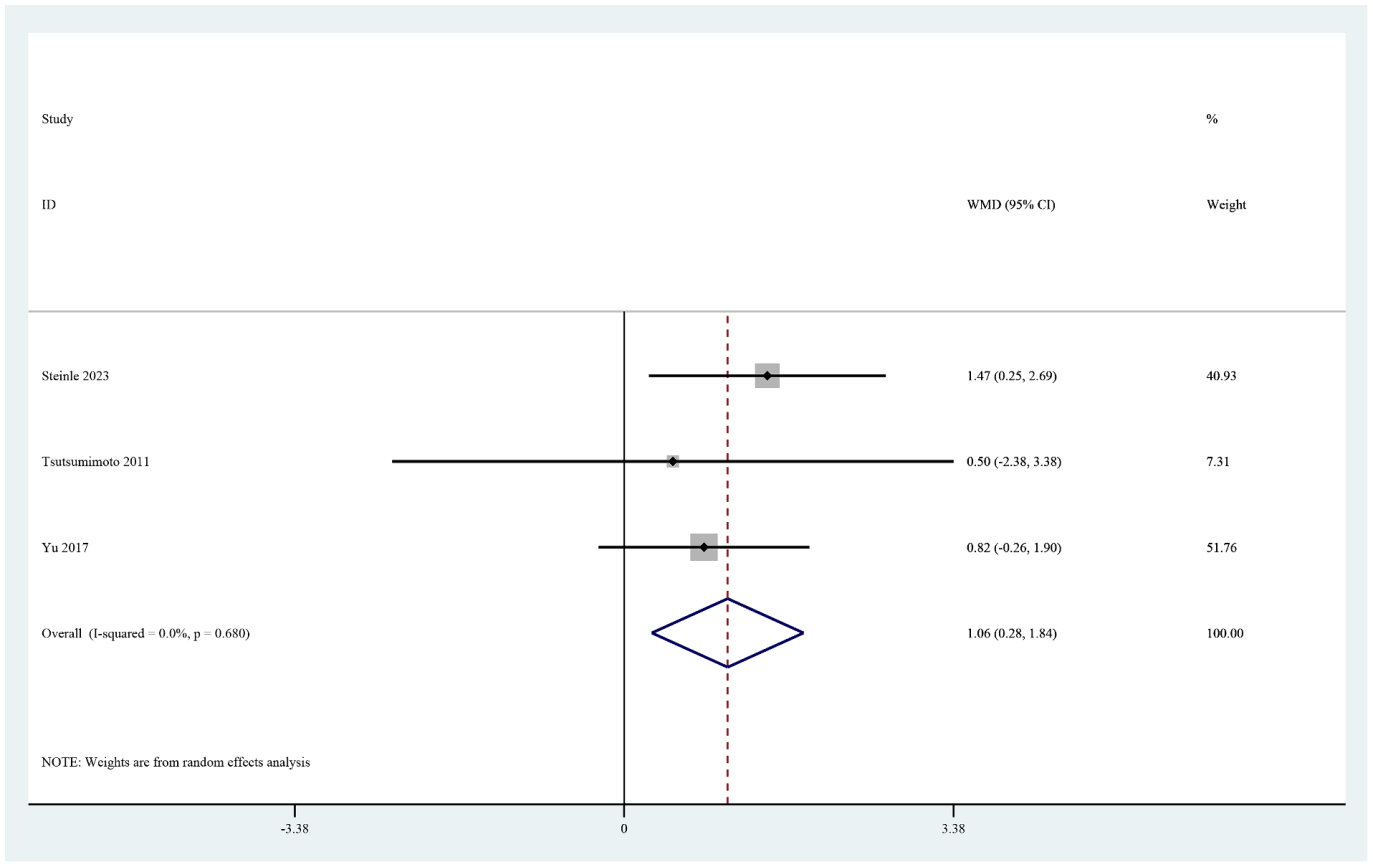
Forest plot of comparison: TXA vs. no-TXA; outcome: HCT levels on the first postoperative day.

The four studies that reported the hemoglobin level on postoperative day 1 (19–21, 26) showed that it was significantly higher in the TXA group than in the control group (MD 0.46, 95% CI 0.21–0.72, I^2^ = 0.0%, *p* < 0.0001; Figure 5). Three of these studies also reported the hematocrit level on postoperative day 1 (19–21). The hematocrit level was significantly higher in the TXA group than in the control group (MD 1.06, 95% CI 0.28–1.84, I^2^ = 0.0%, *p* < 0.0001; Figure 6).

### Complications

**Figure 7:**
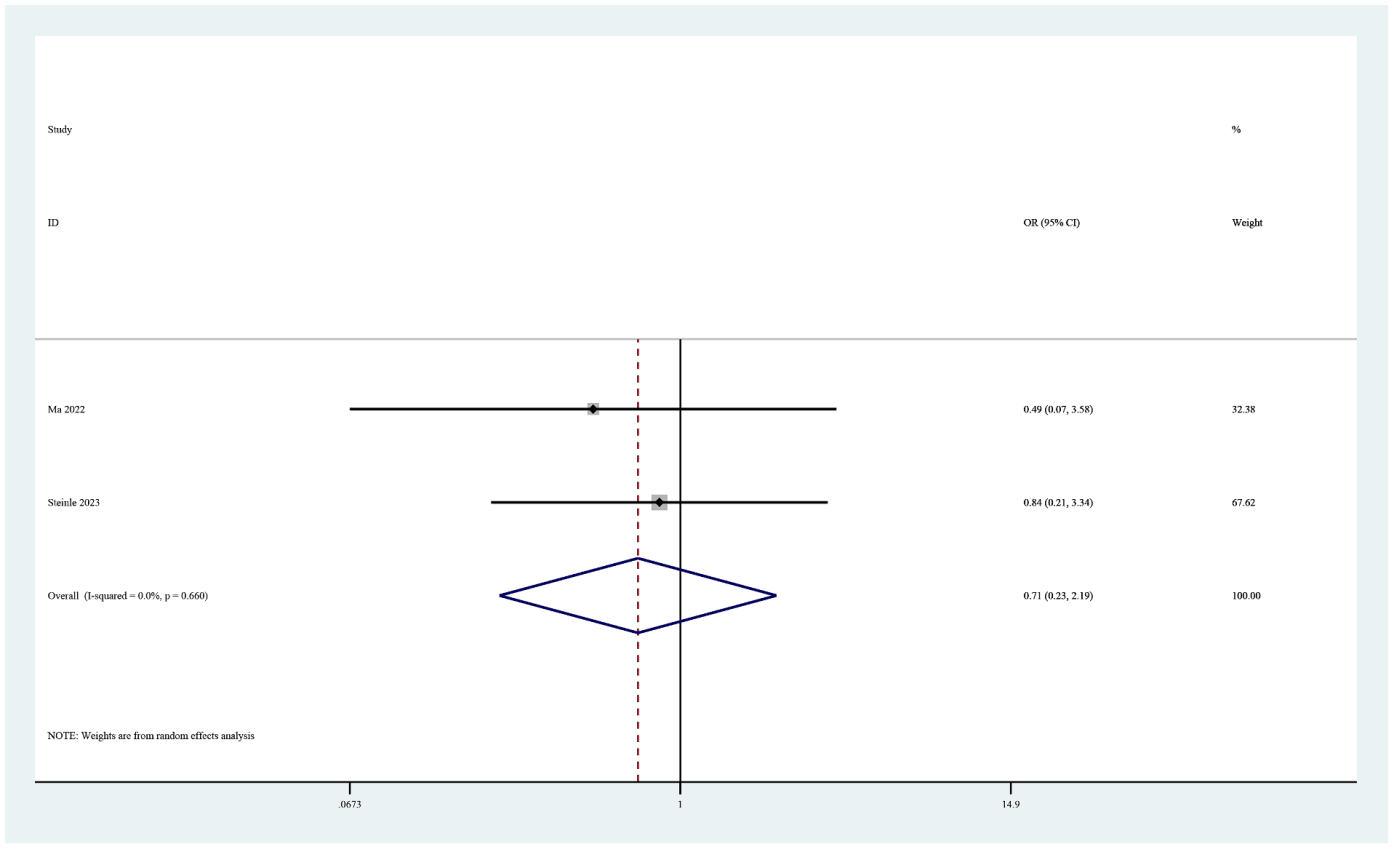
Forest plot of comparison: TXA vs. no-TXA; outcome: Complications.

Eight studies reported complications (19–26). Six of these studies found no drug-related adverse events in either study group (19, 20, 22, 24–26). The pooled results showed no statistically significant difference in postoperative complications between the groups (OR 0.71, 95% CI 0.23–2.19, I^2^ = 0%, *p* = 0.55; Figure 7).

### Sensitivity analysis

The remaining studies were combined when any individual study was excluded. No individual study had a significant effect on the results.

### Risk of bias

Given that fewer than 10 trials were included, no publication bias assessment by funnel plots was performed.

## Discussion

Many individual studies and meta-analyses have highlighted the antifibrinolytic and hemostatic properties of TXA in patients undergoing spinal surgery (27–30). TXA is often associated with reduced intraoperative and postoperative blood loss and may even reduce the need for blood transfusions. However, there have been no meta-analyses of the use of TXA in cervical spine surgery. In this study, we conducted a meta-analysis of eight studies that included a total of 932 patients to estimate the effect of TXA in cervical surgery. Only two of these studies were RCTs that provided high-quality evidence. The main advantages of RCTs are their ability to minimize bias and make stronger causal inferences. The studies randomly assigned participants and controlled for confounding factors, which makes their conclusions more reliable. However, our study also included six trials that were not randomized or controlled. Cumulatively, our inclusion of non-RCT, each achieving NOS scores of 8 or higher, underscores their high research quality. This, in turn, bolsters our ability to furnish robust and trustworthy evidence to substantiate our conclusions. The pooled results showed significant reductions in intraoperative blood loss, total blood loss, postoperative drainage, and loss of hemoglobin and hematocrit on the first postoperative day in comparison with the control group. When patients undergo surgery, there is a transient increase in fibrinolysis, which is thought to be a precipitating factor for blood loss during spinal surgery (31). TXA functions as a competitive antagonist of lysine binding sites on plasminogen, plasmin, and tissue plasminogen. This reversible blockade hinders fibrinolysis and degradation of blood clots and is activated intraoperatively and immediately after surgery, thereby reducing bleeding (32, 33). A study of 7,331 trauma patients by Knowlton et al. (34), a study of 168 knee arthroplasty procedures by Xue et al. (35), and a posterior spinal surgery study by Luan et al. (10) showed that use of TXA did not increase the risk of venous thrombosis in the lower extremities. In our pooled results, there were five cases of thrombosis-related complications in the TXA group and nine in the control group, with no marked difference between the two groups. Luo et al. found that use of TXA in spine surgery may induce epilepsy (36), and the incidence of convulsions increased from 0.5–1.0% to 6.4–7.3% with administration of TXA 50–100 mg/kg in cardiac surgery (37). No convulsion-related complications were reported in the studies included in our meta-analysis and review.

### Limitations

This research had some limitations. First, most of the results were obtained under conditions of high heterogeneity, suggesting that there may be significant differences between studies, possibly owing to differences in patient populations, surgical procedures performed, doses of TXA, route of administration, publication bias, and potentially other unknown factors. Second, there were differences in the dose of TXA and its route of administration in the different studies. For example, Chen et al. injected 1 g of TXA around the wound while Ho et al. administered 10 mg/kg of TXA intravenously (25). Other studies applied topical TXA by irrigation, while others used soaked sponges, and some used local injection (24, 26). Therefore, the consistency and interpretability of our results may be affected by multiple TXA doses and regimens. Third, there were differences between the participants in the different studies, which may have introduced limitations in terms of heterogeneity, bias, and generalization. Again, these differences may affect the interpretation and applicability of the results of pooled analyses, so more rigorous statistical analysis and interpretation are needed to ensure confidence and clinical utility. Despite these limitations, our study provides strong evidence for the role of TXA in cervical surgery. However, more research is needed to address these limitations and confirm our findings.

## Conclusion

The current evidence indicates that TXA significantly reduces blood loss in cervical surgery. Furthermore, TXA was confirmed not to increase the risk of complications. However, there was heterogeneity between studies and some outcomes differed across the different studies. Therefore, there is a need for future studies to examine the effects of TXA in different populations and types of surgery in greater detail to gain a more complete understanding of the role and applicability of TXA in cervical surgery.

## Data availability statement

The original contributions presented in the study are included in the article/supplementary material, further inquiries can be directed to the corresponding author.

## Author contributions

HL: Conceptualization, Data curation, Formal analysis, Funding acquisition, Investigation, Methodology, Validation, Visualization, Writing – original draft. YY: Data curation, Formal analysis, Writing – original draft. ZW: Supervision, Validation, Writing – original draft. LM: Supervision, Writing – review & editing. CX: Conceptualization, Data curation, Formal analysis, Investigation, Methodology, Writing – review & editing.
